# Physical and mental health of breast cancer patients and survivors before and during successive SARS-CoV-2-infection waves

**DOI:** 10.1007/s11136-023-03400-6

**Published:** 2023-04-04

**Authors:** Claudia A. Bargon, Dieuwke R. Mink van der Molen, Marilot C. T. Batenburg, Lilianne E. van Stam, Iris E. van Dam, Inge O. Baas, Liesbeth M. Veenendaal, Wiesje Maarse, Maartje Sier, Ernst J. P. Schoenmaeckers, Josephina P. J. Burgmans, Rhodé M. Bijlsma, Femke van der Leij, Annemiek Doeksen, Danny A. Young-Afat, Helena M. Verkooijen

**Affiliations:** 1 Division of Imaging and Oncology, University Medical Centre Utrecht, Cancer Centre, Heidelberglaan 100, 3584 CX Utrecht, The Netherlands; 2 Department of Surgery, St. Antonius Hospital, Utrecht, The Netherlands; 3 Department of Radiation Oncology, University Medical Centre Utrecht, Cancer Centre, Utrecht, The Netherlands; 4 Department of Medical Oncology, University Medical Centre Utrecht, Cancer Centre, Utrecht, The Netherlands; 5 Department of Surgery, Alexander Monro Hospital, Bilthoven, The Netherlands; 6 Department of Plastic, Reconstructive and Hand Surgery, University Medical Centre Utrecht, Utrecht, The Netherlands; 7 Department of Surgery, Rivierenland Hospital, Tiel, The Netherlands; 8 Department of Surgery, Meander Medical Centre, Amersfoort, The Netherlands; 9 Department of Surgery, Diakonessenhuis Utrecht, Utrecht, The Netherlands; 10 Department of Plastic, Reconstructive and Hand Surgery, Amsterdam University Medical Centre, Amsterdam, The Netherlands; 11 Utrecht University, Utrecht, The Netherlands

**Keywords:** Breast cancer, COVID-19, Mental health, Psychosocial well-being, Corona virus, SARS-CoV-2, Longitudinal, Pandemic, Patient-reported outcomes

## Abstract

**Purpose:**

During the first SARS-CoV-2-infection wave, a deterioration in emotional well-being and increased need for mental health care were observed among patients treated or being treated for breast cancer. In this follow-up study, we assessed patient-reported quality of life (QoL), physical functioning, and psychosocial well-being during the second SARS-CoV-2-infection wave in a large, representative cohort.

**Methods:**

This longitudinal cohort study was conducted within the prospective, multicenter UMBRELLA breast cancer cohort. To assess patient-reported QoL, physical functioning and psychosocial well-being, COVID-19-specific surveys were completed by patients during the first and second SARS-CoV-2-infection waves (April and November 2020, respectively). An identical survey was completed by a comparable reference population during the second SARS-CoV-2-infection waves. All surveys included the validated EORTC-QLQ-C30/BR23, HADS and “De Jong-Gierveld Loneliness” questionnaires. Pre-COVID-19 EORTC-QLQ-C30/BR23 and HADS outcomes were available from UMBRELLA. Response rates were 69.3% (*n* = 1106/1595) during the first SARS-CoV-2-infection wave and 50.9% (*n* = 822/1614) during the second wave. A total of 696 patients responded during both SARS-CoV-2-infection waves and were included in the analysis comparing patient-reported outcomes (PROs) during the second SARS-CoV-2-infection wave to PROs during the first wave. Moreover, PROs reported by all patients during the second SARS-CoV-2-infection wave (*n* = 822) were compared to PROs of a similar non-cancer reference population (*n* = 241) and to their pre-COVID-19 PROs.

**Results:**

Patient-reported QoL, physical functioning, and psychosocial well-being of patients treated or being treated for breast cancer remained stable or improved from the first to the second SARS-CoV-2-infection wave. The proportion of emotional loneliness reduced from 37.6 to 29.9% of patients. Compared to a similar non-cancer reference population, physical, emotional, and cognitive functioning, future perspectives and symptoms of dyspnea and insomnia were worse in patients treated or being treated for breast cancer during the second SARS-CoV-2-infection wave. PROs in the second wave were similar to pre-COVID-19 PROs.

**Conclusion:**

Although patients scored overall worse than individuals without breast cancer, QoL, physical functioning, and psychosocial well-being did not deteriorate between the first and second wave. During the second wave, PROs were similar to pre-COVID-19 values. Overall, current findings are cautiously reassuring for future mental health of patients treated or being treated for breast cancer.

**Supplementary Information:**

The online version contains supplementary material available at 10.1007/s11136-023-03400-6.

## Introduction

The World Health Organization declared the coronavirus disease 2019 (COVID-19) outbreak a pandemic on March 11, 2020 [1, 2]. Due to the immediate high burden on healthcare systems, adapted cancer treatment protocols were implemented rapidly [3–11]. A subsequent temporary disruption in breast cancer screening programs[12, 13], changed referral patterns and changed attitudes toward health care consumption [3, 4, 14] contributed to a sharp decrease in breast cancer diagnoses in 2020 [12, 15]. In response to the dropping cancer diagnoses and abrupt changes in cancer care, increased concerns and anxiety about the consequences of delay and discontinuation of screening, diagnosis, and treatment were observed among cancer patients [2, 14, 16–18].

The unpredictable course of the viral spread still provides an uncertain prospect toward the future for patients [19, 20]. Despite governmental efforts, including social restrictions and rapid development of COVID-19 vaccines, the global pandemic continues to affect daily life through mutant variants of the virus [21]. It is estimated that the current pandemic is likely to last for years, and that social restrictions should not be discarded completely before 2024 due to possible resurgence in contagion [22].

Regardless of a pandemic, patients with currently active or a history of breast cancer have an increased risk of impaired mental health compared to individuals without cancer [23, 24]. As long-term consequences of adverse mental health among breast cancer patients may result in poorer treatment adherence, impaired prognosis and survival, and higher barriers to returning to work [25–29], the pandemic could result in unforeseen long-term adverse effects [30].

During the first months of the COVID-19 pandemic, a deterioration in emotional well-being and an increased need for mental health care were already observed among breast cancer patients [3, 4, 8, 31]. In our previous study [3], conducted amidst the first SARS-CoV-2-infection wave, we observed a substantial drop in emotional and social functioning among breast cancer survivors, and one in two experienced loneliness. However, initial emotional response could stabilize or diminish after patients adjust to a certain situation [32]. As previously described during the 2009 H1N1 pandemic, psychological effects of a viral pandemic can last until 30 months after its onset [33]. Therefore, it is important to understand and monitor the long-term effects of the lingering COVID-19 pandemic on physical and mental health in patients with breast cancer [3, 32, 34].

The aim of this follow-up study was to assess patient-reported quality of life (QoL), physical functioning, and psychosocial well-being in a large prospective cohort of breast cancer patients during the second SARS-CoV-2-infection wave. For context, we compared patient-reported outcomes (PROs) measuring QoL, physical functioning, and psychosocial well-being during the second SARS-CoV-2-infection wave to (1) PROs of the same population during the first SARS-CoV-2-infection wave, (2) PROs of a similar non-cancer reference population, and (3) their own pre-COVID-19 PROs.

## Materials and methods

### Study design and participants

This study was conducted within the prospective, multicenter ‘Utrecht cohort for Multiple BREast cancer intervention studies and Long-term evaluAtion’ (UMBRELLA) [35]. From October 2013 onward, UMBRELLA has been including patients diagnosed with breast cancer in one of six regional hospitals and referred for radiation therapy to the department of Radiation Oncology of the University Medical Center Utrecht. All patients with breast cancer meeting the broad inclusion criteria are invited for participation in UMBRELLA prior to the start of radiation therapy. Inclusion criteria are histologically proven invasive breast cancer or ductal carcinoma in situ (DCIS), having an age ≥ 18 years, sufficient written and spoken understanding of the Dutch language and the absence of mental impairment. All participants provided informed consent for longitudinal collection and use of clinical data and PROs through paper or online questionnaires at regular intervals up to 10 years, i.e., prior to radiation therapy (baseline), after 3 and 6 months, and each 6 months thereafter [35]. Clinical data are routinely provided and updated by the Netherlands Cancer Registry (NCR) [36].

The UMBRELLA study adheres to the Dutch Law on Medical Research Involving Human Subjects (WMO) and the Declaration of Helsinki (version 2013). The study was approved by the Medical Research Ethics Committee (MREC) Utrecht (NL52651.041.15, MEC15/165) and is registered on clinicaltrials.gov (NCT02839863).

### Data collection

UMBRELLA participants were invited to complete two consecutive online COVID-19-specific surveys at the height [37] of the first and second SARS-CoV-2-infection wave in the Netherlands, i.e., 6 weeks and nine months, respectively, after the COVID-19 outbreak on February 27, 2020 [15]. Participants received the first survey on April 7, 2020, and the second survey on November 4, 2020. A reminder was sent after two weeks in case of non-response. Only patients who opted for online questionnaires were eligible for participation in this study. During both peaks, similar governmental restrictions and health care measures, as described in our previous paper [3], were in place in the Netherlands, with the exemption of the national breast cancer screening program, which was resumed two months before the second survey was sent [38].

The COVID-19-specific surveys included three validated questionnaires for the assessment of patient-reported QoL, physical functioning, and psychosocial well-being; the European Organization for Research and Treatment (EORTC) core (C30) and breast cancer-specific (BR23) Quality of Life Questionnaire (QLQ)[39]; Hospital Anxiety and Depression Scale (HADS)[40]; and the De Jong-Gierveld Loneliness Scale [41], complemented by COVID-19-related questions. The COVID-19-related questions were developed by clinical experts and epidemiologists and focused on the presence of COVID-19 and health care consumption (Supplementary Table 1).

Patient-reported QoL, future perspectives, physical, role, emotional, social, and cognitive functioning, symptoms of dyspnoea and insomnia and financial difficulties were assessed with the EORTC-QLQ-C30 and -BR23 questionnaires [39]. For each one to five item subscale, a summary score ranging from 0 to 100 was calculated [42]. Higher scores on the QoL, future perspectives, and functional subscales indicate better outcomes, whereas higher scores on the symptom and financial difficulties subscales indicate worse outcomes. The EORTC-QLQ-C30 and -BR23 scales are considered reliable measures of patient-reported QoL of breast cancer patients [39, 43–45]. Clinical relevance of the mean scores was determined according to previously published thresholds according to EORTC-guidelines, and the clinical relevance of differences between scores were determined according to previously published minimally clinically important differences (MIDs) [46–49].

Symptoms of anxiety and depression were assessed with the 14-item HADS questionnaire, which includes two 7-item subscales for anxiety and depression [40]. Summary scores range from 0 to 21 on each subscale [50]. A higher score represents a higher risk of symptoms of anxiety and/or depression. Scores > 11 on the total HADS scale and scores > 7 on the two subscales represent clinically relevant symptoms of anxiety and/ or depression [51–54]. The HADS has shown a high reliability among different Dutch populations [50]. Breast cancer-specific MIDs for the HADS are yet to be developed [55].

Feelings of loneliness were assessed with the six-item scale of the De Jong-Gierveld Loneliness Scale questionnaire [41]. This short scale consists of two 3-item subscales for emotional loneliness and social loneliness. On the total scale, a score of 0–1 represents the absence of loneliness, 2–4 moderate loneliness and 5–6 severe loneliness [56]. On the subscales, a score of 0–1 indicates no emotional/social loneliness and 2–3 emotional/social loneliness. The De Jong-Gierveld short scales for emotional and social loneliness are considered highly reliable measures for loneliness in the Dutch population [57].

Clinical data, as collected in the context of UMBRELLA or retrieved by the NCR[36], included age at cohort inclusion, sex, body mass index (BMI), highest educational level, type of surgery, (neo-)adjuvant radiation and systemic therapy, and pathological T-stage (AJCC 7th/8th edition).

### Similar reference population without cancer

During the second SARS-CoV-2-infection wave, a similar (i.e., individuals with a similar socio-economic status) and non-cancer reference population was invited to complete all relevant questions of the COVID-19-specific survey. For this purpose, all UMBRELLA participants who received the second COVID-19-specific survey were asked to invite an acquaintance (e.g., friends, colleagues, relatives, or neighbors) to complete a similar online survey. The link to this survey and the accompanying online patient information folder was added to the invitation to the UMBRELLA-participant. This enabled the UMBRELLA-participant to forward this link to the acquaintance. The survey did not include personal identifiable data. All non-cancer individuals who completed the online form provided informed consent to participate. Inclusion criteria for the reference population were as follows: same gender, age within a five-year age range from the participants’ age. Exclusion criteria were a history of or currently diagnosed cancer and completion of < 100% of the survey. Inclusion and exclusion criteria were communicated to the UMBRELLA-participant who forwarded the link and were verified through the survey.

### Pre-COVID-19 PROs

PROs of responders during the second SARS-CoV-2-infection wave were compared to their pre-COVID-19 PROs. The pre-COVID-19 EORTC-QLQ-C30/BR23 and HADS scores were available from UMBRELLA and were derived from regular UMBRELLA questionnaires that were completed in the year before the first COVID-19 diagnosis in the Netherlands on February 27, 2020 [15].

To adjust for differences in follow-up distribution among responders pre-COVID-19 and during the second SARS-CoV-2-infection wave, pre-COVID-19 PROs were weighted for follow-up since cohort inclusion. This is important, as previous studies have shown that physical functioning and psychosocial well-being are expected to gradually improve during the first years after breast cancer diagnosis, independent of a pandemic [3]. Follow-up was divided into four categories, and the following statistical weights were assigned for each subgroup to match the follow-up distribution during the second SARS-CoV-2-infection wave: 0.42 for a follow-up < 1 year, 1.56 for a follow-up between 1–2 years, 1.01 for a follow-up between 3 and 5 years, and 2.99 for a follow-up > 5 years.

### Statistical analysis

Frequencies and proportions, means with range and standard deviation (SD), or medians with range or interquartile range (IQR) were used to describe baseline characteristics of non-responders, responders, and the non-cancer reference population, as well as unadjusted mean EORTC-QLQ-C30/BR23, HADS and De Jong-Gierveld Loneliness Scale scores during the first and second SARS-CoV-2-infection wave.

Three main comparisons are to be distinguished. First, unadjusted mean EORTC-QLQ-C30/BR23 scores were compared between the first and second SARS-CoV-2-infection wave using the paired t-test, unadjusted median HADS scores with the Wilcoxon signed-rank test, the proportion of loneliness on the De Jong-Gierveld Loneliness Scale with the McNemar-Bowker test, and proportions of emotional and social loneliness with the McNemar test. All analyses were performed on complete cases, i.e., those who completed the survey during the first and second SARS-CoV-2-infection wave.

Second, unadjusted mean PROs of all responders during the second SARS-CoV-2-infection wave were compared to unadjusted mean PROs of the non-cancer reference population using the independent samples T-test for the mean EORTC-QLQ-C30/BR23 scores and Mann–Whitney U-test for the median HADS scores. The Chi-square test was used to assess the differences in proportions on the De Jong-Gierveld Loneliness Scale.

Third, mean PROs of responders during the second SARS-CoV-2-infection wave were compared to their weighted mean pre-COVID-19 PROs. Only complete cases on each individual EORTC-QLQ-C30/BR23 and HADS subdomain (i.e., those who completed the survey during the second SARS-CoV-2-infection wave and pre-COVID-19) were compared according to known MIDs [47–49]. Not all subdomains of the pre-COVID-19 questionnaire were completed by each participant. This resulted in varying sample sizes per subdomain of each questionnaire for this comparison.

During the early months of the COVID-19 pandemic, PROs of patients under active treatment were affected more than PROs of patients without active treatment (i.e., those in follow-up) [3]. Therefore, we additionally assessed differences in the proportion of patients with clinically relevant impairment on the different QoL domains in patients with and without active treatment separately between the first and second SARS-CoV-2-infection wave using the McNemar test.

All reported *p*-values were two-sided. Because of multiple testing, *p*-values < 0.01 were considered statistically significant. All statistical analyses were performed using IBM Statistical Package for Social Sciences software, version 26 (SPSS; IBM Corp, Armonk, NY).

## Results

**Fig. 1:**
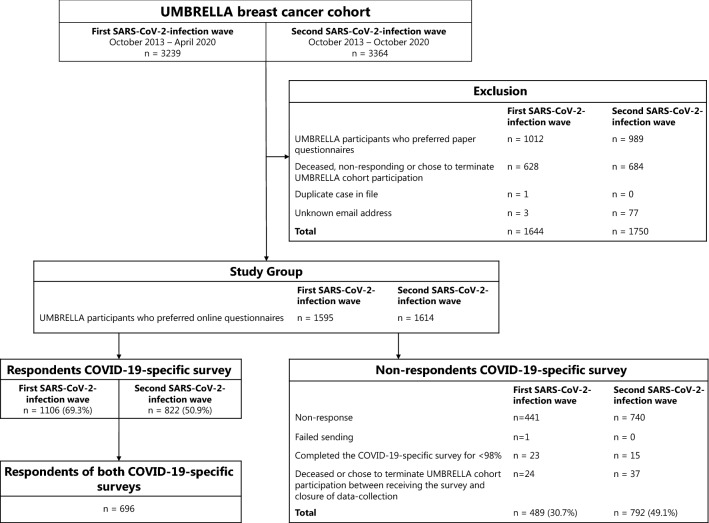
Flowchart of included patients treated or being treated for breast cancer from the prospective, multicenter ‘Utrecht cohort for Multiple BREast cancer intervention studies and Long-term evaluAtion’ (UMBRELLA)

Between October 2013 and April 2020, 3239 patients were enrolled in UMBRELLA (Fig. 1). Of those, 1595 patients were eligible for receiving the COVID-19-specific survey during the first SARS-CoV-2-infection wave. By November 2020, 3364 patients were enrolled in UMBRELLA, of whom 1614 met the inclusion criteria for the second COVID-19-specific survey. Response rates were 69.3% (*n* = 1106) during the first SARS-CoV-2-infection wave, and 50.9% (*n* = 822) during the second SARS-CoV-2-infection wave.

In total, 696 patients responded to both COVID-19-specific surveys (Fig. 1). Mean age of responders to both surveys was 56 years (range = 29–79, SD = 9.2) and mean BMI was 26.1 (SD = 4.7, Table 1). Median follow-up was 31 months (range = 1–85). The majority were diagnosed with a pathological T1-stage tumor (*n* = 401, 57.6%) and treated with breast conserving surgery (*n* = 555, 79.7%) and (loco)regional radiation therapy (*n* = 631, 90.7%). Most responders (*n* = 554, 79.6%) were living with a partner and/or child(ren), and 25.6% (*n* = 178) had received mental healthcare support since diagnosis. During both waves, baseline characteristics of resp

**Table 1:** Baseline characteristics of responders of the COVID-19-specific surveys (*n* = 696) during the first and second SARS-CoV-2-infection waves (i.e., April 2020 and November 2020, respectively) and of the non-cancer reference population (*n* = 241)

	Responders to both COVID-19-specific surveys (*n* = 696)^a^	Non-cancer reference population (*n* = 241)^a^
**Patient characteristics**		
Age in years, mean (range; SD)	56 (29–79; 9.2)	58 (31–82; 9.2)
Sex, No. (%)		
Female	693 (99.6)	228 (94.6)
Male	3 (0.4)	13 (5.4)
Body Mass Index^b^, mean (SD)	26.1 (4.7)	25.7 (4.2)
Missing, No. (%)	3 (0.4)	0 (0.0)
Highest educational level		
Primary or (post-)secondary school	293 (42.1)	98 (40.7)
College, graduate or professional degree	401 (57.6)	143 (59.3)
Unknown	2 (0.3)	0 (0.0)
Current living situation		
With partner and/or child(ren)	554 (79.6)	190 (78.8)
Alone/other	142 (20.4)	51 (21.2)
Follow-up time^c^ in months, median (range)	31 (1–85)	–
Tumor characteristics		
Pathological T-stage, No. (%)		
0 + In situ (IS)	112 (16.1)	–
I	401 (57.6)	–
II–IV	150 (21.6)	–
X + unknown	33 (4.7)	–
**Treatment characteristics**		
Type of breast surgery		
Breast conserving therapy	555 (79.7)	–
Mastectomy ± delayed reconstruction	61 (8.8)	–
Mastectomy with direct breast reconstruction	63 (9.1)	–
None	12 (1.7)	–
Unknown	5 (0.7)	–
Systemic therapy^d^		
No systemic therapy	253 (36.4)	–
Chemotherapy	71 (10.2)	–
Endocrine therapy	135 (19.4)	–
Immunotherapy	0 (0.0)	–
Combination of above^e^	232 (33.3)	–
Unknown	5 (0.7)	–
Radiation therapy		
Yes	631 (90.7)	–
No	42 (6.0)	–
Unknown	23 (3.3)	–
Currently receiving active breast cancer treatment^f^		
Yes	279 (40.1)	–
No	417 (59.9)	–
Supportive care^g^		
Mental support	60 (8.6)	–
Physical or other support	193 (27.7)	–
Mental and physical/other support	118 (17.0)	–
None	325 (46.7)	–
Are / were you infected by the COVID-19?		
Yes, confirmed by nasopharyngeal swab	17 (2.4)	3 (1.2)
Possibly, I have or had fever	31 (4.5)	16 (6.6)
No, I was tested negative	164 (23.6)	67 (27.8)
No, I had/ have no symptoms and I was not tested	484 (69.5)	155 (64.3)

As a result of rounding, percentages may not add up a 100%

*SD* Standard deviation; – not available.

^a^Baseline characteristics of complete cases (i.e., responders to both COVID-19-specific surveys) and the reference population as measured during the second SARS-CoV-2-infection wave. The first SARS-CoV-2-infection wave in the Netherlands was in April 2020 and second SARS-CoV-2-infection wave in November 2020

^b^BMI was calculated as weight(kg)/height(m)^2^ and based on the last measurement in UMBRELLA

^c^Follow-up time was defined as time from cohort inclusion to completion of the COVID-19-specific survey

^d^Pre- and/ or postoperative therapy

^e^Combination of chemotherapy, endocrine therapy and/or immunotherapy

^f^Current active treatment was defined as being treated with chemotherapy, endocrine therapy, immunotherapy and/or radiation therapy at the time of completing the COVID-19-specific survey

^g^Supportive care included physical and/or mental support. Mental support was defined as having received mental support by a psychologist, psychiatrist or coach, having received pastoral care or having contact with peers. Physical or other support is defined as having received physical therapy or oncological rehabilitation or having received therapy by a sexologist or dietitian

onders and non-responders were comparable (Supplementary Table 2).

The non-cancer reference population included 241 individuals without currently active or a history of cancer with a mean age of 58 years (range = 31–82, SD = 9.2, Table 1). The majority (*n* = 190, 78.9%) were living with their partner and/or child(ren). Baseline characteristics of the reference population were similar to the responders of both surveys regarding age, sex, BMI, educational level, and current living situation (Table 1).

The number of responders to the survey during the second SARS-CoV-2-infection wave who had also completed the pre-COVID-19 survey varied per questionnaire and per subdomain, resulting in a population of 664–729 participants depending on the subdomain (Table 4).

### The second vs the first SARS-CoV-2-infection wave

In comparison with the first SARS-CoV-2-infection wave, the mean score for emotional functioning improved from 78.4 (SD = 17.0) to 81.4 (SD = 17.5) in the second wave (*p* < 0.001, Table 2). According to established MIDs [47, 48], these mean differences (MDs) were not clinically meaningful. No other statistically significant or clinically important differences were observed in unadjusted mean EORTC-QLQ-C30/BR23 scores from the first to the second wave.

**Table 2:** PROs of physical functioning and psychosocial well-being of all patients treated or being treated for breast cancer who completed both COVID-19-specific surveys (EORTC-QLQ-C30 and -BR23, HADS and the De Jong-Gierveld) during both SARS-CoV-2-infection waves (*n* = 696)

	First SARS-CoV-2-infection wave^a^	Second SARS-CoV-2-infection wave^a^	*p*-value^c^
EORTC-QLQ-C30 and -BR23^b^	Mean (SD)	Mean (SD)	
Quality of Life (QoL)	79.7 (17.0)	79.4 (15.8)	0.572
Future perspectives (FP)	69.7 (19.8)	70.7 (21.3)	0.190
Functioning scales			
Physical functioning (PF)	89.1 (13.4)	88.4 (14.5)	0.060
Role functioning (RF)	81.9 (23.8)	83.8 (23.3)	0.039
Emotional functioning (EF)	78.4 (17.0)	81.4 (17.5)	**< 0.001**
Social functioning (SF)	88.9 (20.1)	88.3 (19.9)	0.497
Cognitive functioning (CF)	82.8 (17.9)	81.4 (20.6)	0.023
Symptom scales			
Dyspnoea (D)	9.8 (18.2)	10.1 (18.7)	0.689
Insomnia (I)	27.8 (26.9)	28.7 (27.3)	0.363
Financial difficulties (FD)	5.0 (14.3)	5.7 (16.7)	0.138
HADS^d^	Median (IQR)	Median (IQR)	*p*-value^c^
Total	6 (4–10)	5 (2–10)	** < 0.001**
Anxiety	4 (3–6)	3 (1–6)	**< 0.001**
Depression	2 (1–5)	1 (0–4)	** < 0.001**
De Jong-Gierveld Loneliness Scale^e^	*n* (%)	*n* (%)	*p*-value^c^
Not lonely	364 (52.3)	404 (58.0)	0.021
Moderately lonely	273 (39.2)	225 (32.3)	
Severely lonely	59 (8.5)	67 (9.6)	
Emotional loneliness scale			
Not emotionally lonely	434 (62.4)	488 (70.1)	< 0.001
Emotionally lonely	262 (37.6)	208 (29.9)	
Social loneliness scale			
Not socially lonely	572 (82.2)	555 (79.7)	0.135
Socially lonely	124 (17.8)	141 (20.3)	

As a result of rounding, percentages may not add up a 100%

CF Cognitive Functioning; D Dyspnea; EF Emotional Functioning; EORTC European Organization for Research and Treatment of Cancer; FD Financial Difficulties; HADS Hospital Anxiety and Depression Score; I Insomnia; IQR Interquartile Range; PF Physical Functioning; QoL Quality of Life; RF Role Functioning; SF Social Functioning; SD Standard deviation

Bold *p*-values are considered statistically significant (*p* < 0.01).

^a^The first SARS-CoV-2-infection wave in the Netherlands was in April 2020 and second SARS-CoV-2-infection wave in November 2020

^b^EORTC-QLQ-C30 and -BR23 scores range from 0 to 100. Higher scores represent better outcomes for all functioning scales, and lower scores on symptom scales indicate better outcomes

^c^Unadjusted mean EORTC QLQ-C30 and -BR23 scores were compared between the first and second SARS-CoV-2-infection wave using the paired t-test, unadjusted median HADS scores with the Wilcoxon signed-rank test, proportion of Loneliness on the De Jong-Gierveld Loneliness Scale with the McNemar-Bowker test, and the proportions of emotional and social loneliness with the McNemar test

^d^A HADS total score > 7 indicates a possible anxiety disorder or depression and a score > 11 indicates a probable depression or anxiety disorder

^e^The short scale of the Loneliness Scale was developed by De Jong-Gierveld. Higher scores indicate more severe feelings of loneliness

HADS subscores improved 1 point from the first to the second SARS-CoV-2-infection wave (all *p* < 0.001). The proportion of patients feeling emotionally lonely decreased from 37.6% (*n* = 262) to 29.9% (*n* = 208, *p* < 0.001, Table 2). No statistically significant differences were found in social loneliness between the first and second SARS-CoV-2-infection wave.

### Patients vs the non-cancer reference population during the second SARS-CoV-2-infection wave

During the second SARS-CoV-2-infection wave, patients treated or being treated for breast cancer scored statistically significantly worse than the non-cancer and similar reference population on all EORTC-QLQ-C30/BR23 domains, except for financial difficulties (Table 3). When considering known MIDs[47, 48], patient-reported future perspectives (MD = 7.8), physical (MD = 5.0), emotional (MD = 5.2), and cognitive functioning (MD = 9.0), dyspnoea (MD = 5.6), and insomnia (MD = 7.9) seemed to reflect clinically relevant differences, all favoring the non-cancer reference population.

**Table 3:** Mean EORTC-QLQ-C30 and -BR23, median HADS scores, and the proportion of individuals experiencing loneliness according to the De Jong-Gierveld Loneliness Scale during the second SARS-CoV-2-infection wave (*n* = 822) compared to a non-cancer reference population (*n* = 241)

	Responders COVID-19-specific survey (*n* = 822)	Non-cancer reference population (*n* = 241)	*p*-value^c^
	*Second* SARS-CoV-2-infection wave^a^	*Second* SARS-CoV-2-infection wave^a^	
EORTC-QLQ-C30 and -BR23^b^	Mean (SD)	Mean (SD)	
Quality of Life (QoL)	79.1 (16.1)	82.8 (14.5)	0.001
Future perspectives (FP)	70.6 (21.5)	78.4 (21.0)	< 0.001
Functioning scales			
Physical functioning (PF)	88.1 (14.5)	93.1 (11.0)	< 0.001
Role functioning (RF)	83.4 (23.4)	89.3 (18.6)	< 0.001
Emotional functioning (EF)	81.2 (17.6)	86.4 (15.8)	< 0.001
Social functioning (SF)	87.8 (20.4)	92.3 (17.1)	0.001
Cognitive functioning (CF)	81.2 (20.7)	90.2 (16.8)	< 0.001
Symptom scales			
Dyspnoea (D)	10.6 (19.0)	5.0 (13.4)	< 0.001
Insomnia (I)	28.8 (27.7)	20.9 (23.4)	< 0.001
Financial difficulties (FD)	5.5 (16.3)	3.2 (13.7)	0.027
HADS^d^	Median (IQR)	Median (IQR)	*p*-value^c^
Total	5 (2–10)	4 (2–8)	0.114
Anxiety	3 (1–6)	3 (1–5)	0.062
Depression	1 (0–4)	1 (0–4)	0.528
De Jong-Gierveld Loneliness Scale^e^	*n* (%)	*n* (%)	*p*-value^f^
Not lonely	482 (58.6)	131 (54.4)	0.068
Moderately lonely	261 (31.8)	94 (39.0)	
Severely lonely	79 (9.6)	16 (6.6)	
Emotional loneliness scale			
Not emotionally lonely	581 (70.7)	152 (63.1)	0.025
Emotionally lonely	241 (29.3)	89 (36.9)	
Social loneliness scale			
Not socially lonely	660 (80.3)	208 (86.3)	0.034
Socially lonely	162 (19.7)	33 (13.7)	

As a result of rounding, percentages may not add up a 100%

*CF* Cognitive Functioning, *D* Dyspnea; *EF* Emotional Functioning, *EORTC* European Organization for Research and Treatment of Cancer, *FD* Financial Difficulties, *HADS* Hospital Anxiety and Depression Score, *I* Insomnia, *IQR* Interquartile Range, *PF* Physical Functioning; *QoL* Quality of Life, *RF* Role Functioning, *SF* Social Functioning; *SD* Standard deviation; – not available, bold *p*-values are considered statistically significant (*p* < 0.01).

^a^The second SARS-CoV-2-infection wave in the Netherlands was in November 2020. Pre-COVID-19 was defined the period of one year before the first COVID-19 diagnosis in the Netherlands (i.e., 27th of February, 2020)

^b^EORTC-QLQ-C30 and -BR23 scores range from 0 to 100. Higher scores represent better outcomes for all functioning scales, and lower scores on symptom scales indicate better outcomes

^c^Unadjusted mean PROs of patients during the second SARS-CoV-2-infection wave were compared to unadjusted mean PROs of the non-cancer reference population using the independent samples T-test for the mean EORTC-QLQ-C30 and -BR23 scores, and the Mann–Whitney U-test for the median HADS scores

^d^A HADS total score > 7 indicates a possible anxiety disorder or depression and a score > 11 indicates a probable depression or anxiety disorder

^e^The short scale of the Loneliness Scale was developed by De Jong-Gierveld. Higher scores indicate more severe feelings of loneliness

^f^The Chi-square test was used to assess differences in proportions on the De Jong-Gierveld Loneliness Scale among patients and non-cancer reference individuals during second SARS-CoV-2-infection wave

No statistically significant differences were found between patients and the reference population regarding anxiety and depression (HADS), nor regarding the proportion experiencing social, emotional, or overall loneliness.

### The second SARS-CoV-2-infection wave vs pre-COVID-19

When compared to pre-COVID-19, emotional functioning (MD = 3.2) showed the largest deterioration during the second SARS-CoV-2-infection wave (Table 4). None of the MDs for the EORTC-QLQ-C30/BR23 subdomains seemed of clinical importance.

**Table 4:** Mean EORTC-QLQ-C30 and -BR23 and median HADS scores during the second SARS-CoV-2-infection wave (*n* = 664–729), compared to weighted mean pre-COVID-19 scores (*n* = 664–729)

	Responders COVID-19-specific survey (*n* = 664–729^a^)	Responders COVID-19-specific survey (*n* = 664–729^a^)
	*Pre-COVID-19*^b^	*Second* SARS-CoV-2-infection wave^b^
EORTC-QLQ-C30 and -BR23^c^	Weighted mean^d^ (SD)	Mean (SD)
Quality of Life (QoL)	78.7 (17.7)	79.7 (15.8)
Future perspectives (FP)	74.5 (23.0)	71.6 (20.9)
Functioning scales		
Physical functioning (PF)	88.2 (14.8)	88.5 (14.4)
Role functioning (RF)	81.9 (23.8)	84.5 (22.6)
Emotional functioning (EF)	85.0 (17.5)	81.8 (17.3)
Social functioning (SF)	89.2 (19.5)	89.0 (19.5)
Cognitive functioning (CF)	82.1 (20.2)	81.7 (20.4)
Symptom scales		
Dyspnoea (D)	10.5 (19.3)	10.3 (18.8)
Insomnia (I)	25.2 (26.4)	27.9 (27.2)
Financial difficulties (FD)	4.7 (14.4)	5.4 (16.3)
HADS^e^	Weighted median^d^ (IQR)	Median (IQR)
Total	6 (3–10)	5 (2–9)
Anxiety	4 (2–6)	3 (1–6)
Depression	2 (1–4)	1 (0–4)

As a result of rounding, percentages may not add up a 100%

*CF* Cognitive Functioning, *D* Dyspnea, *EF* Emotional Functioning, *EORTC* European Organization for Research and Treatment of Cancer, *FD* Financial Difficulties, *HADS* Hospital Anxiety and Depression Score, *I* Insomnia, *IQR* Interquartile Range, *PF* Physical Functioning, *QoL* Quality of Life, *RF* Role Functioning; *SF* Social Functioning, *SD* Standard deviation

not available, bold *p*-values are considered statistically significant (*p* < 0.01)

^a^Number of responders to the survey during the second wave who also completed pre-COVID-19 responses per subdomain: QoL *n* = 726; FP *n* = 725; PF *n* = 729; RF *n* = 728; EF *n* = 726; SF *n* = 726; CF *n* = 726; D *n* = 727; I *n* = 727; FD *n* = 725; HADS total *n* = 692; HADS anxiety *n* = 663; HADS depression *n* = 664

^b^The second SARS-CoV-2-infection wave in the Netherlands was in November 2020. Pre-COVID-19 was defined the period of one year before the first COVID-19 diagnosis in the Netherlands (i.e., 27th of February, 2020). In case responders completed > 1 regular UMBRELLA questionnaires in the year before COVID-19, the most recently completed questionnaire was used

^c^EORTC-QLQ-C30 and -BR23 scores range from 0 to 100. Higher scores represent better outcomes for all functioning scales, and lower scores on symptom scales indicate better outcomes

^d^Mean PROs of patients during the second SARS-CoV-2-infection wave were compared to weighted mean/median PROs pre-COVID-19 using the EORTC-QLQ-C30 and -BR23 scores, and the HADS scores. PROs were weighted for follow-up since cohort inclusion (follow-up < 1 year: 0.42, follow-up 1–2 years: 1.56, follow-up 3–5 years: 1.01, follow-up > 5 years: 2.99)

^e^A HADS total score > 7 indicates a possible anxiety disorder or depression and a score > 11 indicates a probable depression or anxiety disorder

In comparison to pre-COVID-19, the HADS showed one point decrease in all subdomains during the second wave.

### Patients with clinically relevant impairment of PROs during the first and second SARS-CoV-2-infection wave

When comparing the first to the second SARS-CoV-2-infection wave, no statistically significant differences were observed in the proportion of patients with clinically relevant impairment of PROs (data not shown). Also, in stratified analyses by active or no active treatment at the time of completing the COVID-19-specific surveys, no statistically significant differences in the proportion of patients with clinically relevant impairment of PROs were found (Supplementary Table 3). Overall, when comparing patients with (*n* = 175) and without (*n* = 417) active treatment, patients with active treatment showed higher proportions of clinically relevant impairment on all EORTC-QLQ-C30/BR23 and HADS domains than patients without active treatment during both SARS-CoV-2-infection waves.

## Discussion

This large prospective observational study observed several clinically important and reassuring findings concerning the impact of the ongoing COVID-19 pandemic on patient-reported QoL, physical functioning, and psychosocial well-being among breast cancer patients up to nine months since onset. From the first to the second SARS-CoV-2-infection wave, emotional functioning, and symptoms of anxiety and depression improved, while all other QoL scores remained stable. Also, the proportion of patients experiencing emotional loneliness decreased from 37.6 to 29.9%. However, during the second SARS-CoV-2-infection wave, patients treated or being treated for breast cancer still scored worse on all QoL domains in comparison with a similar non-cancer reference population of similar age, except for financial difficulties. When comparing PROs during the second SARS-CoV-2-infection wave to pre-COVID-19 PROs, outcomes were largely similar.

In accordance with our findings, previous studies observed a worldwide increase in anxiety and depression among breast cancer patients and survivors during the first months of the pandemic [31, 34, 58–60]. Fortunately, we observed no further clinically meaningful deterioration on all QoL-subdomains, anxiety or depression among breast cancer patients nine months after the onset of the pandemic. Moreover, the proportion of patients with clinically relevant impairment on the different QoL-subdomains did not change statistically significantly from the first to the second SARS-CoV-2-infection wave.

Emotional functioning and emotional loneliness improved between the first and second SARS-CoV-2-infection wave, and lower levels of anxiety and depression were observed. Independent of a pandemic, gradual increases in physical functioning and psychosocial well-being and QoL are generally observed over time during the first years after diagnosis among patients treated or being treated for breast cancer [3, 61]. However, as mean follow-up of our study population was 31 months, our cohort was likely to have reached a plateau in which this improvement in mental well-being has naturally stabilized [62–64]. Moreover, whether emotional functioning (MD = 3.0) improved to a clinically relevant extent is debatable. A small MID has previously been estimated at MD > 5–10[49, 63, 65]; however, a breast cancer-specific MID for emotional functioning has yet to be established [47, 48].

Similar to our findings, Rentscher and colleagues [66] found an increase in loneliness among breast cancer survivors during the first months of the pandemic in comparison with pre-COVID-19, and no difference in loneliness compared to matched controls. Likewise, a Dutch study [24] observed increased loneliness among cancer patients and their family members during the first SARS-CoV-2-infection wave. From the first to the second SARS-CoV-2-infection wave, we observed a decrease in the proportion of patients reporting moderate to severe overall loneliness from 47.7 to 42.0%, which was also comparable to our non-cancer reference population. Nonetheless, these proportions are still substantially higher than the previously published 34% of individuals experiencing loneliness in a general Dutch population of comparable age in 2019, pre-COVID-19 [67].

COVID-19 has an especially large impact on the mental health of individuals with currently active or a history of cancer when compared to similar and non-cancer reference individuals [68]. A large American study showed that, shortly after the COVID-19 outbreak, cancer survivors were more likely to feel anxious, depressed and lonely than adults without cancer [69]. Nine months after the onset of the pandemic patients still scored worse than the non-cancer reference population on all QoL-subdomains, however, we did not observe differences in anxiety, depression, and loneliness. Based on known MIDs [47, 48], the observed differences for patient-reported QoL, financial difficulties, social, role, and physical functioning would be considered clinically trivial, and differences for emotional and cognitive functioning, dyspnea and insomnia of small clinical relevance [47, 48]. No breast cancer-specific MID has been established yet for the subdomain future perspectives [47, 48], but based on the established MID for future perspectives among multiple myeloma patients, an MD < 10 would not be considered clinically meaningful [70]. Several pre-COVID-19 studies showed that, regardless of a pandemic, breast cancer patients have a higher risk of worse mental health and cognitive issues than non-cancer individuals [23, 71]. Therefore, the observed differences with small clinical relevance are most likely explained by the treatment of the disease and/or the disease itself rather than the COVID-19 pandemic.

When compared to pre-COVID-19, PROs during the second SARS-CoV-2-infection wave provided reassuring perspective, as all observed differences in EORTC-QLQ-C30/BR23 scores between pre-COVID-19 and the second wave were estimated clinically trivial [47, 49, 70]. No breast cancer-specific MIDs for the HADS have been established yet [55], however, based on the MIDs for the HADS among patients with chronic obstructive pulmonary disease[72], the differences in HADS scores would also not be considered clinically meaningful. Thus, the previously observed deterioration in psychosocial well-being during the first SARS-CoV-2-infection [3] wave could likely have reflected a temporal effect of the pandemic, as most QoL domains seemed to return to pre-COVID-19 levels during the second SARS-CoV-2-infection wave. As such, it seems that participants adjusted to the new situation and learned to live through a pandemic.

To date, herd immunity, effectiveness of vaccines and SARS-CoV-2-infection control measures have improved, resulting in dropping hospitalization and mortality rates due to SARS-CoV-2-infection [73, 74]. While this has likely reduced levels of anxiety toward contracting SARS-CoV-2-infection, the pandemic continues to cause concerns about its long-term effects. For example, the effectiveness of vaccines beyond six months is still unclear [73], and it has been reported that women older than 20 years, and thus, the vast majority of breast cancer patients are more likely to develop at least one out of the three ‘Long COVID’ symptoms, including persistent fatigue with bodily pain or mood swings, ongoing respiratory problems, or cognitive problems [75].

Since the start of the pandemic, there has been extra attention for the mental health of breast cancer patients through the implementation of various e-mental-health projects [76–78]. Although improved over time, emotional functioning of breast cancer patients was still lower during the second wave than pre-COVID-19 and lower when compared to non-cancer individuals. Consequences of decreased emotional functioning should not be underestimated. Impaired mental health in breast cancer patients is associated with poorer treatment compliance and might thereby negatively affect survival [29, 34, 79]. As such, long-term effects of the current COVID-19 pandemic on mental health of breast cancer patients should continue to be monitored to minimize adverse effects of the pandemic in the future [16, 68, 79, 80]. Moreover, previous studies have shown that social isolation and the lack of emotional support from family and friends are associated with higher risk of mortality [81, 82]. For current clinical practice and beyond an apparent end of the pandemic, it is therefore strongly advised to (further) develop and implement e-mental health applications and psychosocial interventions, aiming to improve mental health of this vulnerable population that is reported to be uniquely at risk for experiencing emotional distress [2, 31].

This study has some limitations. First, although baseline characteristics of responders and non-responders were comparable during both infection waves, an under- or overestimation of the results due to selective (non-)response could not be ruled out as the reasons for non-response were unclear. Second, only participants who had opted to complete online surveys were eligible for this study. Participants who opted for paper surveys were excluded, possibly resulting in some degree of selection bias. Third, although baseline characteristics, and thus, socio-economic status, of the reference population were similar to our UMBRELLA participants, the reference population might still have been subject to some degree of selection bias, as all reference individuals were directly or indirectly faced with the impact of breast cancer diagnosis and treatment through the related UMBRELLA-participant. Last, the results of this study represent PROs during the first and second SARS-CoV-2-infection peaks in the Netherlands and might therefore be subject to fluctuations that follow the severity of the infection peaks. An important strength of this study is that this is the first longitudinal study to evaluate the course of PROs in patients treated or being treated for breast cancer from pre-COVID-19 until the second SARS-CoV-2-infection wave. The ongoing collection of clinical data and PROs within UMBRELLA allows for future analyses with successive PROs during and after consecutive SARS-CoV-2-infection waves to monitor the impact of the pandemic and its subsequent social measures on physical functioning and psychosocial well-being of breast cancer patients and survivors, aiming to support nationwide preventive and curative mental health care programs.

## Conclusions

Despite the lingering COVID-19 pandemic, patient-reported QoL, physical functioning and psychosocial well-being in individuals treated or being treated for breast cancer did not deteriorate between the first and second SARS-CoV-2-infection waves. Emotional functioning, anxiety, and depression slightly improved, and the proportion of patients experiencing emotional loneliness decreased. Compared to a similar and non-cancer reference population, individuals treated or being treated for breast cancer still scored clinically meaningfully worse for patient-reported physical, emotional, and cognitive functioning, future perspectives, dyspnea, and insomnia nine months after the COVID-19 outbreak. However, in comparison with pre-COVID-19, no clinically meaningful differences were found during the second SARS-CoV-2-infection wave.

## Supplementary Information

Below is the link to the electronic supplementary material.

Supplementary file1 (DOCX 54 KB)

## Data Availability

The data underlying this article, including individual de-identified participant data and a data dictionary defining each field in the set, will be shared on reasonable request to the corresponding author.

## Declarations

### Originality of the work

This manuscript, including related data, figures and tables, is solely the work of the authors stated, has not been published, and is not under consideration for publication elsewhere.
