# Supplementary material for: Physical and mental health of breast cancer patients and survivors before and during successive SARS-CoV-2-infection waves
Source: Qual Life Res. 2023 Apr 4;32(8):2375–90. doi: 10.1007/s11136-023-03400-6 (PMC10072805; doi:10.1007/s11136-023-03400-6)
Supplement: Supplementary file 1 — Supplementary file1 (DOCX 54 KB) [file 11136_2023_3400_MOESM1_ESM.docx]

**Supplementary Tables**

**Supplementary Table 1** The COVID-19-related questions as developed by our clinical experts and epidemiologists.

**Supplementary Table 2** Baseline characteristics^a^ of responders and non-responders of the COVID-19-specific surveys during the first and second SARS-CoV-2-infection waves of the COVID-19 pandemic.

**Supplementary Table 3** Comparison of proportions of responders with clinically relevant impairment of PROs on the EORTC-QLQ30 and -BR23 and HADS questionnaires between both SARS-CoV-2-infection waves, stratified into patients receiving active treatment (n=175) and patients receiving no active treatment, i.e., follow-up care (n=417).

**Supplementary Table 1 The COVID-19-related questions as developed by our clinical experts and epidemiologists.**

| **Are / were you infected by the COVID-19?^a, b^** | 🞏 Yes, confirmed by nasopharyngeal swab  🞏 Possibly, I have or had fever, but I did not test it^d^  🞏 No, I was tested negative^d^  🞏 No, I had/ have no symptoms and I was not tested^d^  🞏 No^c^  Explanation: … |
| --- | --- |
| **If you have been infected, what were your most severe symptoms?^a, b^** | 🞏 No symptoms  🞏 Mild symptoms^c^  🞏 Moderate symptoms^c^  🞏 Severe symptoms^c^  🞏 No hospital admission, a cold end/or coughing and/or sore throat^d^  🞏 No hospital admission, but loss of taste and/or smell^d^  🞏 No hospital admission, but fever and/or dyspnea^d^  🞏 Hospital admission  🞏 Intensive care admission  Explanation: … |
| **What are your current symptoms?^a^** | 🞏 No symptoms  🞏 Mild symptoms  🞏 Moderate symptoms  🞏 Severe symptoms  🞏 Hospital admission  🞏 Intensive care admission  Explanation: … |
| **If you have not been infected, are you worried you might get infected?^a, b^** | 🞏 Not at all  🞏 A little bit  🞏 Quite a bit  🞏 A lot  Explanation: … |
| **What situation corresponds best to your current situation?^a, b^** | 🞏 My treatment has not started yet^d^  🞏 I currently receive radiotherapy or chemotherapy for breast cancer^c^  🞏 I currently receive radiotherapy^d^  🞏 I currently receive chemotherapy^d^  🞏 I currently receive hormonal therapy^c^  🞏 I currently receive hormone therapy or endocrine therapy^d^  🞏 I currently receive immune therapy (trastuzumab / Herceptin®)^d^  🞏 I currently do not receive active treatment, but a ‘wait-and-see’ treatment or ‘’active surveillance’’^d^  🞏 I currently do not receive any active treatment anymore, but I am still in follow-up  🞏 I currently do not receive any active treatment anymore, I also finished follow-up^d^  🞏 Other, namely |
| **Do the current COVID-19 measure affect your current treatment or (after)care?^a, b^** | 🞏 Yes  🞏 No  🞏 I don’t know^d^  Explanation: … |
| **Which effects did you experience during COVID-19 regarding your breast cancer treatment or (supportive) care so far?^b^** | 🞏 None  🞏 One or more treatments/consultations/medical examinations in the hospital were cancelled  🞏 One or more treatments/consultations/medical examinations in the hospital were postponed  🞏 One or more consultations/medical examinations in the hospital were changed into a digital/telephone consultation  🞏 The treatment was adjusted  🞏 The treatment was postponed  🞏 The treatment was halted  🞏 A second opinion was postponed  🞏 A second opinion was cancelled  🞏 Other, namely  Explanation: … |
| **Do you expect that the current COVID-19 measures will affect your treatment or (after)care in the future?^a,b^** | 🞏 Yes  🞏 No  🞏 I don’t know^d^  Explanation: … |
| **Did the threshold to contact your general practitioner change, because of the COVID-19 situation?^a,b^** | 🞏 Yes, I contact my general practitioner more easily  🞏 Yes, I contact my general practitioner less easily  🞏 No, but during the first COVID-19 wave I contacted my general practitioner less easily^d^  🞏 No  Explanation: … |
| **Did the threshold to contact the physicians treating your breast cancer change, because of the COVID-19 situation?^a, b^** | 🞏 Yes, I contact my breast cancer physician(s) more easily  🞏 Yes, I contact my breast cancer physician(s) less easily  🞏 No, but during the first COVID-19 wave I contacted my physicians less easily^d^  🞏 No  Explanation: … |
| **Did the threshold to discuss your breast cancer diagnosis or breast cancer (treatment) related symptoms with family and friends change, because of the COVID-19 situation?^a, b^** | 🞏 Yes, I contact my friends and family more easily  🞏 Yes, I contact my friends and family less easily  🞏 No, but during the first COVID-19 wave I contacted my family and friends less easily^d^  🞏 No  Explanation: … |
| **Are you worried about your financial situation as a result of COVID-19?^a, b^** | 🞏 Not at all  🞏 A little bit  🞏 Quite a bit  🞏 Very much  Explanation: … |
| **Did you have contact with a physician since the start of the COVID-19 outbreak in the Netherlands (27^th^ of February 2020)?^b^** | 🞏 Yes, with my physician treating my breast cancer  🞏 Yes, with my general practitioner  🞏 No, this was not necessary  🞏 No, but I would have wanted this |
| **Which of the following supportive (after)care did you receive since you received your diagnosis breast cancer?**  **Multiple answer options are possible.^b^** | 🞏 None  🞏 Psychiatrist  🞏 Psychologist  🞏 Coaching  🞏 Peer-contact  🞏 Dietician  🞏 Sexologist  🞏 Physiotherapist  🞏 Oncological Rehabilitation  🞏 Pastoral care  🞏 Other, namely… |
| **Since the start of the COVID-19 outbreak, medical and/or supportive (after)care has not always been physically possible. Did you feel the need for digital alternatives, so-called ‘’e-(mental) health’’? For example, you could think of digital programs or consultations with a dietician, coach, or online peer-contact.^b^** | 🞏 Yes, also when physical meetings are possible, digital alternatives could be good  🞏 Yes, digital options are a good alternative in case physical meetings are not possible  🞏 Maybe  🞏 No, I would only want to receive (after)care through physical meetings  🞏 Other, namely... |

^a^ This question was included in the COVID-19-specific questionnaire of the first SARS-CoV-2-infection wave.

^b^ This question was included in the COVID-19-specific questionnaire of the second SARS-CoV-2-infection wave.

^c^ This response option was only included in the COVID-19-specific questionnaire of the first SARS-CoV-2-infection wave.

^d^ This response option was only included in the COVID-19-specific questionnaire of the second SARS-CoV-2-infection wave.

**Supplementary Table 2 Baseline characteristics^a^ of responders and non-responders of the COVID-19-specific surveys during the first and second SARS-CoV-2-infection wave of the COVID-19 pandemic.**

|  | **Responders (n=1106, 69.3%)** | | **Non-responders (n=489, 30.7%)** | | **Responders (n=822, 50.9%)** | | **Non-responders (n=49.1%)** |  |  |
| --- | --- | --- | --- | --- | --- | --- | --- | --- | --- |
|  |  |  |  |  |  |  |  |  |  |
|  | **First SARS-CoV-2-infection wave^b^** | | | | **Second SARS-CoV-2-infection wave^b^** | | |  |  |
| **Patient characteristics** |  | |  | |  | |  |  |  |
| Age in years, mean (range) | 56 (24-82) | | 55 (27-92) | | 57 (29-83) | | 55 (24-92) |  |  |
| Sex, No. (%) |  | |  | |  | |  |  |  |
| Female | 1100 (99.5) | | 487 (99.6) | | 819 (99.6) | | 788 (99.5) |  |  |
| Male | 6 (0.5) | | 2 (0.4) | | 3 (0.4) | | 4 (0.5) |  |  |
| Body Mass Index^c^, mean (SD) | 26.1 (4.7) | | 26.4 (5.1) | | 26.1 (4.7) | | 26.3 (4.9) |  |  |
| Missing, No. (%) | 11 (1.0) | | 42 (8.6) | | 45 (5.5) | | 72 (9.1) |  |  |
| Highest educational level |  | |  |  |  | |  |  |  |
| Primary or (post-)secondary school | 488 (44.1) | | 225 (46) | | 339 (41.2) | | 349 (44.1) |  |  |
| College, graduate or  professional degree | 609 (55.1) | | 222 (45.4) | | 439 (53.5) | | 372 (47.0) |  |  |
| Unknown | 9 (0.8) | | 42 (8.6) | | 44 (5.4) | | 71 (9.0) |  |  |
| Current living situation |  | |  |  |  | |  |  |  |
| With partner and/or child(ren) | - | | - | | 647 (78.8) | | - |  |  |
| Alone/other | - | | - | | 175 (21.3) | | - |  |  |
| Follow-up time in months, median (range) | 29 (1-78) | | 32 (1-78) | | 33 (1-85) | | 37 (1-85) |  |  |
| **Tumor characteristics** |  | |  | |  | |  |  |  |
| Pathological T stadium, No. (%) |  | |  |  |  |  |  |  |  |
| 0 + In situ (IS) | 173 (15.6) | | 91 (18.6) | | 121 (14.7) | | 134 (16.9) |  |  |
| I | 641 (58.0) | | 264 (54) | | 442 (53.8) | | 416 (52.5) |  |  |
| II-IV | 238 (21.5) | | 112 (22.9) | | 178 (21.6) | | 171 (21.6) |  |  |
| X + unknown | 54 (4.9) | | 22 (4.5) | | 81 (9.9) | | 71 (9.0) |  |  |
| **Treatment characteristics** |  |  |  |  |  |  |  |  |  |
| Type of breast surgery |  | |  | |  | |  |  |  |
| Breast conserving therapy | 863 (78.0) | | 393 (80.4) | | 619 (75.3) | | 582 (73.5) |  |  |
| Mastectomy ± delayed  reconstruction | 106 (9.6) | | 39 (8.0) | | 69 (8.4) | | 76 (9.6) |  |  |
| Mastectomy with direct breast | 109 (9.9) | | 46 (9.4) | | 69 (8.4) | | 84 (10.6) |  |  |
| reconstruction |  |  |  |  |  |  |  |  |  |
| None | 20 (1.8) | | 9 (1.8) | | 14 (1.7) | | 13 (1.6) |  |  |
| Unknown | 8 (0.7) | | 2 (0.4) | | 51 (6.2) | | 37 (4.7) |  |  |
|  |  | |  | |  | |  |  |  |
|  | **Responders (n=1106, 69.3%)** | | **Non-responders (n=489, 30.7%)** | | **Responders (n=822, 50.9%)** | | **Non-responders (n=49.1%)** |  |  |
|  |  | |  | |  | |  |  |  |
|  | **First SARS-CoV-2-infection wave^b^** | | | | **Second SARS-CoV-2-infection wave^b^** | | |  |  |
| **Treatment characteristics** |  | |  | |  | |  |  |  |
| Systemic therapy^d^ |  | |  | |  | |  |  |  |
| No systemic therapy | 410 (37.1) | | 172 (35.2) | | 278 (33.8) | | 282 (35.6) |  |  |
| Chemotherapy | 100 (9.0) | | 53 (10.8) | | 77 (9.4) | | 72 (9.1) |  |  |
| Endocrine therapy | 201 (18.2) | | 99 (20.2) | | 155 (18.9) | | 130 (16.4) |  |  |
| Immunotherapy | 0 (0.0) | | 1 (0.2) | | 0 (0.0) | | 1 (0.1) |  |  |
| Combination of above^e^ | 387 (35.0) | | 162 (33.1) | | 261 (31.8) | | 270 (34.1) |  |  |
| Unknown | 8 (0.7) | | 2 (0.4) | | 51 (6.2) | | 37 (4.7) |  |  |
| Radiation therapy |  | |  | |  | |  |  |  |
| Yes | 993 (89.8) | | 438 (89.6) | | 702 (85.4) | | 672 (84.8) |  |  |
| No | 77 (7.0) | | 38 (7.8) | | 47 (5.7) | | 62 (7.8) |  |  |
| Unknown | 36 (3.3) | | 13 (2.7) | | 73 (8.9) | | 58 (7.3) |  |  |
| Currently receiving active breast cancer treatment^f^ |  | |  | |  | |  |  |  |
| Yes | 350 (31.6) | | - | | 269 (32.7) | | - |  |  |
| No | 756 (68.4) | | - | | 553 (67.3) | | - |  |  |
| Supportive care^g^ |  | |  | |  | |  |  |  |
| Mental support | - | | - | | 67 (8.2) | | - |  |  |
| Physical or other support | - | | - | | 224 (27.3) | | - |  |  |
| Mental and physical/other  support | - | | - | | 128 (15.6) | | - |  |  |
| None | - | | - | | 403 (49.0) | | - |  |  |
| Are / were you infected by the COVID-19? |  | |  | |  | |  |  |  |
| Yes, confirmed by  nasopharyngeal swab | 1 (0.1) | | - | | 21 (2.6) | | - |  |  |
| Possibly, I have or had fever | 103 (9.3) | | - | | 35 (4.3) | | - |  |  |
| No, I was tested negative | 11 (1.0) | | - | | 183 (22.3) | | - |  |  |
| No, I had/ have no symptoms  and I was not tested | 991 (89.6) | | - | | 583 (70.9) | | - |  |  |

As a result of rounding, percentages may not add up a 100%.

*Abbreviations*: SD Standard deviation.

Legend: - Not available.

^a^ Baseline characteristics of responders and non-responders of the first SARS-CoV-2-infection wave may differ from previously published data^3^, as clinical data were routinely updated by the Netherlands Cancer Registry, and the number of responses to the first COVID-19-specific survey increased after analyzing the first responses (due to the timely subject, only responses received before April 24, 2020 were used for analyses in our previous publication^8^).

^b^ The first SARS-CoV-2-infection wave in the Netherlands was in April 2020 and second SARS-CoV-2-infection wave in November 2020.

^c^ Calculated as weight/height^2^.

^d^ Pre- and/ or postoperative therapy.

^e^ Combination of chemotherapy, endocrine therapy and/or immunotherapy.

^f^ Active treatment was defined as receiving chemotherapy, endocrine therapy, immunotherapy and/or radiation therapy at the time of completing the COVID-19-specific survey.

^g^ Mental support was defined as having received mental support by a psychologist, psychiatrist or coach, having received pastoral care or having contact with peers. Physical or other support is defined as having received physical therapy or oncological rehabilitation or having received therapy by a sexologist or dietitian.

**Supplementary Table 3 Comparison of proportions of responders with clinically relevant impairment of PROs on the EORTC-QLQ30 and -BR23 and HADS questionnaires between both SARS-CoV-2-infection waves, stratified into patients receiving active treatment (n=175) and patients receiving no active treatment, i.e., follow-up care (n=417).**

|  | **Clinically relevant threshold scores** | **Active treatment^a^** | | | **No active treatment** | | |
| --- | --- | --- | --- | --- | --- | --- | --- |
|  |  | **(n=175)** | | | **(n=417)** | | |
|  |  | **First SARS-CoV-2-infection wave^b^** | **Second SARS-CoV-2-infection wave^b^** |  | **First SARS-CoV-2-infection wave^b^** | **Second SARS-CoV-2-infection wave^b^** |  |
|  |  | **n (%)** | **n (%)** | **p-value** | **n (%)** | **n (%)** | **p-value** |
| **EORTC-QLQ C30 and -BR23^c^** | | | | | | | |
| *Functioning scales* |  |  |  |  |  |  |  |
| Physical functioning (PF) | < 83 | 46 (26.3) | 49 (28.0) | 0.720 | 74 (17.7) | 79 (18.9) | 0.590 |
| Role functioning (RF) | < 58 | 35 (20.0) | 29 (16.6) | 0.392 | 40 (9.6) | 33 (7.9) | 0.419 |
| Emotional functioning (EF) | < 71 | 52 (29.7) | 53 (30.3) | 1.000 | 109 (26.1) | 91 (21.8) | 0.085 |
| Social functioning (SF) | < 58 | 18 (10.3) | 19 (10.9) | 1.000 | 18 (4.3) | 20 (4.8) | 0.851 |
| Cognitive functioning (CF) | < 75 | 70 (40.0) | 79 (45.1) | 0.163 | 111 (26.6) | 114 (27.3) | 0.818 |
| *Symptom scales* |  |  |  |  |  |  |  |
| Dyspnea (D) | > 17 | 44 (25.1) | 49 (28.0) | 0.511 | 103 (24.7) | 103 (24.7) | 1.000 |
| Insomnia (I) | > 50 | 37 (21.1) | 43 (24.6) | 0.392 | 62 (14.9) | 63 (15.1) | 1.000 |
| Financial difficulties (FD) | > 17 | 30 (17.1) | 31 (17.7) | 1.000 | 45 (10.8) | 42 (10.1) | 0.749 |
| **HADS^d^** | | | | | | | |
| Total | > 11 | 45 (25.7) | 39 (22.3) | 0.392 | 80 (19.2) | 72 (17.3) | 0.403 |
| Anxiety | > 7 | 33 (18.9) | 33 (18.9) | 1.000 | 64 (15.3) | 59 (14.1) | 0.609 |
| Depression | >7 | 21 (12.0) | 19 (10.9) | 0.824 | 41 (9.8) | 38 (9.1) | 0.749 |

As a result of rounding, percentages may not add up a 100%.

*Abbreviations:* CF Cognitive Functioning; D Dyspnea; EF Emotional Functioning; EORTC European Organization for Research and Treatment of Cancer; FD Financial Difficulties; HADS Hospital Anxiety and Depression Score; I Insomnia; PF Physical Functioning; RF Role Functioning; SF Social Functioning.

^a^ Active treatment was defined as receiving chemotherapy, endocrine therapy, immunotherapy and/or radiation therapy at the time of completing the second COVID-19-specific questionnaire in November 2020.

^b^ The first SARS-CoV-2-infection wave in the Netherlands was in April 2020 and second SARS-CoV-2-infection wave in November 2020

^c^ EORTC-QLQ-C30 and -BR23 scores range from 0 to 100.

^d^ A HADS total score >7 indicates a possible anxiety disorder or depression and a score >11 indicates a probable depression or anxiety disorder.
